# Enhancement of Bottle Gourd Oil Activity via Optimized Self-Dispersing Lipid Formulations (SDLFs) to Mitigate Isoproterenol-Evoked Cardiac Toxicity in Rats via Modulating BMP, MMP2, and miRNA-21 and miRNA-23a Genes’ Expression

**DOI:** 10.3390/molecules28166168

**Published:** 2023-08-21

**Authors:** Shereen S. El-Mancy, Sylvia A. Boshra, Osama S. Elnahas, Sahar M. Fayez, Nermin M. Sheta

**Affiliations:** 1 Department of Pharmaceutics, Faculty of Pharmacy, October 6 University, Giza 12585, Egypt; shereenelmancy@o6u.edu.eg (S.S.E.-M.); osamaelnahas@o6u.edu.eg (O.S.E.); saharmfayez@o6u.edu.eg (S.M.F.); nerminsheta@o6u.edu.eg (N.M.S.); 2 Department of Biochemistry, Faculty of Pharmacy, October 6 University, Giza 12585, Egypt

**Keywords:** bottle gourd oil, self-dispersing lipid formulations, BNP, MMP2, miRNAs

## Abstract

Bottle gourd (BG) oil (family Cucurbitaceae) has several pharmacological activities including a reduction of the hazard of cardiovascular and atherosclerosis conditions. This work aimed to develop and optimize self-dispersing lipid formulations (SDLFs) of BG oil by applying a full 3^2^ factorial design. The formulation variables (oil concentration and surfactant mixture ratio) showed an obvious impact on the characters of the prepared BG-SDLFs including droplet size (DS), polydispersity index (PDI), emulsification time (ET), and transmission percentage (Tr%). The optimum BG-SDLF composed of 30% oil and Tween 80/Cremophor^®^ RH40 (1:1) showed good emulsification characteristics and a better drug release profile compared with BG oil. In vivo study in isoproterenol-injected rats showed that BG oil and the optimized BG-SDLF improved cardiac function, by elevating the miRNA-23a gene expression level and decreasing miRNA-21 gene expression. They also caused the inhibition of the plasma B-type natriuretic peptide (BNP), N-terminal proatrial natriuretic peptide (NT-pro-BNP), cystatin c, galectin-3, lipoprotein-associated phospholipase A2 (Lp-PLA2), matrix metallopeptidase 2 (MMP2), cardiac troponin I (cTnI), and cardiac troponin T (cTnT). Our study demonstrated that BG oil and the optimized BG-SDLF provided a cardioprotection against isoproterenol-induced cardiac toxicity with better results in groups treated with the optimized BG-SDLF.

## 1. Introduction

Cardiovascular diseases (CVDs) are the main reason for mortality in the world. Approximately 17.9 million people died from CVDs in 2019, accounting for 32% of all global deaths, [1]. Heart attacks and strokes were responsible for eighty-five percent of these deaths [1]. Myocardial fibrosis is a common side effect of cardiac remodeling that can cause heart failure and death. Myocardial fibrosis is caused by increased myofibroblast activity and excessive extracellular matrix deposition [2]. This process involves a variety of cells and chemicals that could be used as therapeutic targets in the future [2].

Isoproterenol (ISO) is an artificial catecholamine and beta-adrenergic agonist, which causes a boosted oxidative stress in the myocardium, leading to infarctlike death of the cardiac muscle. It produces extremely cytotoxic free radicals through autooxidation, accelerating the peroxidation of phospholipids and causing severe damage to the heart membrane [3].

Bottle gourd (BG) (*Lageneria siceraria*), from the Cucurbitaceae family, is a climbing plant cultivated in tropical countries as a vegetable crop, including Thailand, Egypt, India, and Japan [4,5]. Members of the Cucurbitaceae family are also known as gourds, melons, or pumpkins [6] and are found in various sizes and shapes. Bottle gourd could be found as small or large, rounded or bottle-shaped [5]. Conventional uses of the fruit include cardioprotective [7], antidotal, diuretic, immunosuppressive, and aphrodisiac uses, as well as uses as a free radical scavenger thought to be rich in vitamin B and C and hepatoprotective [8], adaptogenic, stress-reducing, and anti-inflammatory uses [9]. It is also used for its hypolipidemic, antihyperglycemic, antihypertensive, and laxative properties and promotes weight loss due to its dietary fiber content [6]; it is a central nervous system stimulant [5], an analgesic, and a general tonic. The results of previous experiments revealed that *L. siceraria* has an obvious anticancer activity, which could be attributed to its cytotoxicity and antioxidant properties.

BG seeds contain a high concentration of phytochemicals, vitamins, minerals, amino acids, and fatty acids, promoting a precursor of protein, lipids, micro- and macronutrients. According to the literature, BG seed oil contains a high concentration of fatty acids and sterolic entities. It has numerous health benefits, which may be attributed to its elevated content of polyunsaturated fatty acids (PUFA) such as linoleic and linolenic acids, which reduce the risk of CVDs through oxidation resistance [4].

More than forty percent of the newly discovered chemical entities have low solubility in water and complicated formulation issues. They pose significant constraints to develop suitable formulations to increase biological efficacy. For lipophilic drugs with low solubility and high permeability, dissolution is the rate-limiting step in oral absorption. The most common approach to improve their bioavailability is to incorporate the active lipophilic component into lipid vehicles [10].

In general, emulsified formulations, such as self-dispersing lipid formulations (SDLFs), are rapidly absorbable, ensuring a rapid transportation into the circulation. The SDLFs are made up of oil + surfactant mixture + drug. When they combine with an aqueous environment, they form emulsions, which are small droplets of oil dispersed in water. The drug is rapidly distributed throughout the gastrointestinal tract in these fine droplets [10,11]. SDLFs are classified into two types: self-emulsifying drug delivery systems (SEDDS) formed with an HLB less than 12, and self-microemulsifying drug delivery systems (SMEDDS) formed with an HLB greater than 12. SEDDS and SMEDDS are both stable preparations that improve drug dissolution due to an increased surface area on dispersion [12].

To our knowledge, limited studies have been performed for the formulation and in vivo assessment of BG oil and its formulation. The current work aimed to assess the potential of SDLFs as a promising delivery system to overcome formulation challenges of BG oil owing to its hydrophobic/lipophilic properties. Different methods were performed for the characterization of the prepared BG-SDLFs, and an in vivo study was carried out to investigate both BG oil and its optimized SDLF role in the cardioprotective activity against ISO-induced cardiac toxicity.

## 2. Results and Discussion

### 2.1. Experimental Design and Statistical Analysis

Nine BG-SDLFs were prepared based on a 3^2^ full factorial design; the predetermined dependent variables were measured and are recorded in Table 1. The data were statistically analyzed for various models. The two factors interaction (2FI) model was fitted for all studied responses based on the significant *p*-value (*p*-value < 0.05). For each response, the model validation was verified by the value of the actual model’s R^2^ (>0.9), the plausible agreement of the predicted and the adjusted R^2^ values, i.e., the difference was <0.2, and an adequate precision (>4).

#### Effect of Formulation Variables on BG-SDLFs Characteristics

**Figure 1:**
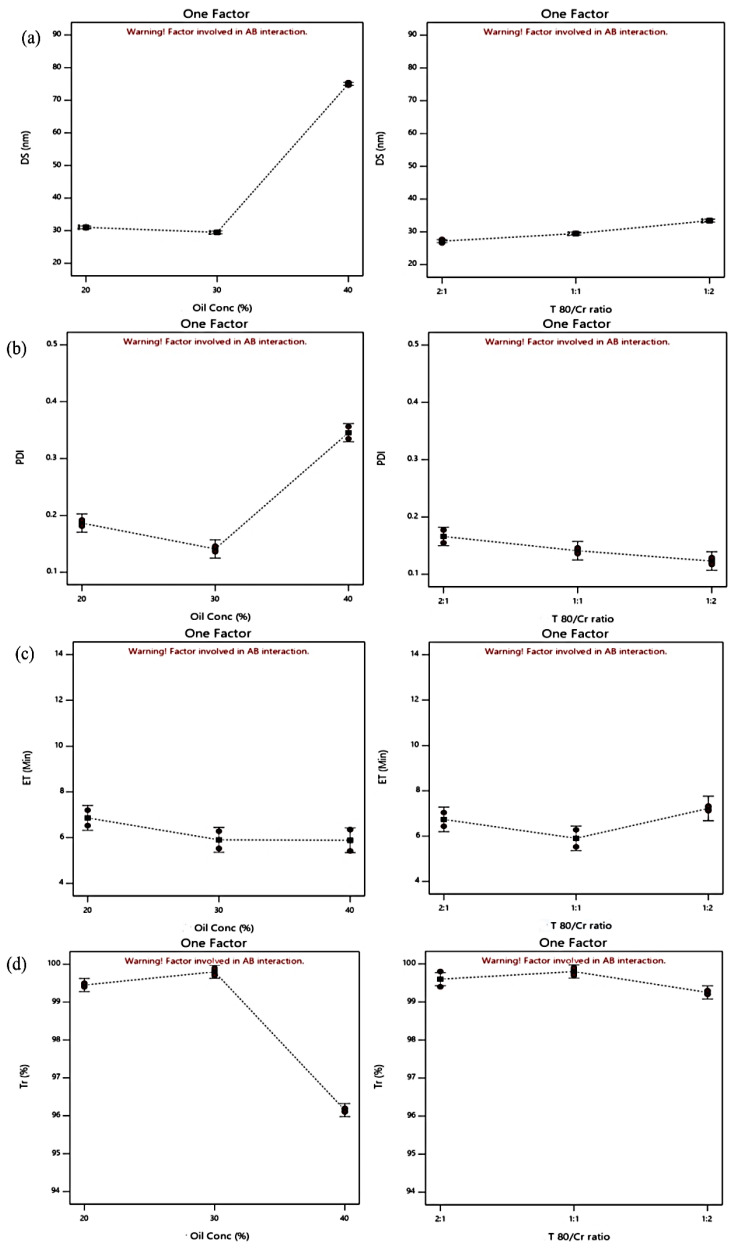
Linear correlation plots presenting the effects of oil concentration (at a Tween 80/Cremophor^®^ RH40 ratio of 1:1) and Tween 80/Cremophor^®^ RH40 ratio (at 30% oil) on (**a**) DS, (**b**) PDI, (**c**) ET, and (**d**) Tr%.

**Figure 2:**
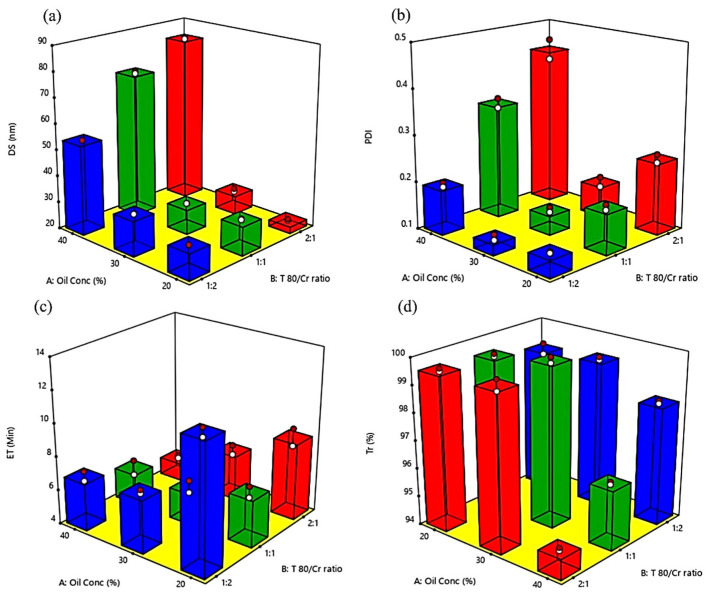
Three-dimensional plots for the effect of compositions of BG-SDLFs (oil concentration and Tween 80/Cremophor^®^ RH40 ratio) on (**a**) DS, (**b**) PDI, (**c**) ET, and (**d**) Tr%.

**Figure 3:**
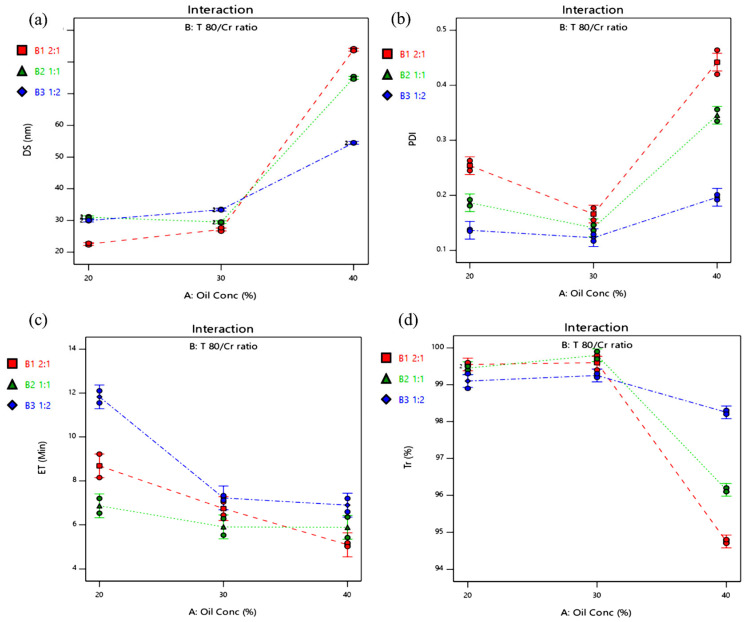
Interactions plots for the effect of compositions of BG-SDLFs on (**a**) DS, (**b**) PDI, (**c**) ET, and (**d**) Tr%.

The effects of the oil concentration (X_1_) and the Tween 80/Cremophor^®^ RH40 ratio (X_2_) are illustrated in Figure 1, Figure 2 and Figure 3 showing linear correlation plots, 3D plots, and the interactions plots for all responses, respectively. The DS is a remarkable factor in SDLFs as it affects the rate and extent of drug release as well as its absorption and bioavailability [13]. DS values of the prepared BG-SDLFs were in the range of 22.6 to 84.3 nm as recorded in Table 1; they reflected the validity of the used formulations to produce BG oil nano-dispersions. The ANOVA analysis report for the DS validated the model with an R^2^ of 0.999 and a reasonable agreement of the predicted R^2^ (0.993), and the adjusted R^2^ (0.997). The oil concentration (X_1_), Tween 80/Cremophor^®^ RH40 ratio (X_2_), and X_1_ X_2_ had significant effects (*p* < 0.001) on the DS. As shown in Figure 1a and Figure 2a, the DS decreased by decreasing the oil concentration; this is obviously due to the corresponding increase in the surfactant mix concentration which produces a smaller DS [14]. As the surfactant concentration increases, the interfacial tension at the oil–water interface decreases, and the surfactant molecules provide a mechanical barrier to coalescence resulting in an increased spontaneity of the nanoemulsion formation, and consequently, smaller dispersions droplets are formed [15]. Regarding the Tween 80/Cremophor^®^ RH40 ratio (X_2_), the smallest DS value was formed at a Tween 80/Cremophor^®^ RH40 ratio of 2:1 of the lower Cremophor^®^ RH40 content; this could be attributed to a lower HLB value, the branched structure, and a relative bulkiness of Cremophor^®^ RH40 molecules leading to an increase in the size of the formed droplets [16]. However, the highest DS was formed for dispersions of the same ratio and 40% oil; this could be explained by the interaction between the tested factors (Figure 3a), as the emulsification is influenced by the formulation composition, HLB value, molecular structure, and chain length of the surfactant [17,18].

PDI expresses the uniformity of the droplet sizes within each formulation dispersion; values less than 0.5 are generally accepted [19]. All PDI values confirmed the good homogeneity of the formed dispersions (they ranged from 0.129 to 0.435), and they were significantly affected by the tested factors as depicted in Figure 1b and Figure 2b.

The ET and Tr % of the prepared BG-SDLFs presented vital indicators for the measurement of the emulsification efficiency. For ET, the model validity was confirmed by an R^2^ of 0.969 and a reasonable agreement of the predicted R^2^ (0.874) and the adjusted R^2^ (0.941). Figure 1c and Figure 2c illustrate that increasing oil concentration caused a decrease in ET, the lowest ET ((5.2 min) was recorded for F7, which was composed of 40% oil and Tween 80/Cremophor^®^ RH40 (2:1). Upon dilution with the aqueous phase, various mesomorphic phases can be formed between the formulation and the water, which may affect both DS and the turbidity of the dispersion [20]. The ease of emulsification could be associated with the ease by which water penetrates the liquid crystalline phase formed on the surface of the droplet [10]. As the surfactant mixture content increases, especially with a higher Cremophor^®^ RH40 content, the formed liquid crystalline phase becomes more viscous and leads to retard self-dispersion, reflecting a difficulty in water penetration of the gel phase [17,21]. In addition, surfactants with higher HLB values were observed to have a higher solubility and better emulsification ability, and Tween 80 showed a good emulsification ability, which allowed a rapid dispersion in previous studies [13,22,23]. Tr% is presented in Figure 1d and Figure 2d; the measured values ranged from 99.8 to 94.8%, and the statistical model was verified by R^2^, predicted R^2^, and adjusted R^2^ values of 0.996, 0.984, and 0.992, respectively. Tr% values more than 90 indicate a good emulsification [14]. We found that these Tr% values matched the DS values; the lowest value was recorded for F7 with the highest DS.

The interactions plots (Figure 3) revealed an interaction between the two investigated formulation factors; thus, the effect of each factor on the tested responses was dependent on the other. The smallest DS was formed at 20% oil and a Tween 80/Cremophor^®^ RH40 ratio of 2:1, the minimum PDI was found at 30% oil and a Tween 80/Cremophor^®^ RH40 ratio of 1:2, while the lowest ET and the highest Tr% were obtained at 30% oil and a Tween 80/Cremophor^®^ RH40 ratio of 1:1.

### 2.2. Formulation Optimization

The optimized BG-SDLF was determined using the desirability function to minimize DS, PDI, ET, and maximize Tr% simultaneously. The optimized BG-SDLF (F5: composed of 30% oil and a Tween 80/Cremophor^®^ RH40 ratio of 1:1) had a desirability value of 0.916 indicating that the formulation fulfilled the maximum desired requirements perfectly. The predicted responses for the optimized formulation were 29.5 nm, 0.141, 5.9 min, and 99.8% for DS, PDI, ET, and Tr%, respectively (Table 1). The similarity between these predicted values and the observed values of F5 (29.4 nm, 0.136, 6.2 min, and 99.8% for DS, PDI, ET, and Tr%, respectively) confirmed the validity of the used design model.

### 2.3. Characterization of the Optimized BG-SDLF

#### 2.3.1. Visual Inspection, Drug Content, and Thermodynamic Stability

A visual detection provides information about the clarity and homogeneity of the prepared formulation. The optimized BG-SDLF showed a clear yellowish appearance tied to the inherent system composition; Tween 80 and Cremophor^®^ RH40 beside the BG oil. The percentage of BG oil in the optimized BG-SDLF was 99.00 ± 1.05%. After both centrifugation and freezing–thawing cycle tests, there were no changes in physical appearance concerning syneresis or phase separation, which indicates a good physical stability.

#### 2.3.2. Robustness to Dilution and Phase Separation

The optimized BG-SDLF did not show any separation or precipitation at the end of the 2 h after the used dilution folds in the various media. The formed dispersions were clear and transparent.

#### 2.3.3. In Vitro Dissolution Studies

**Figure 4:**
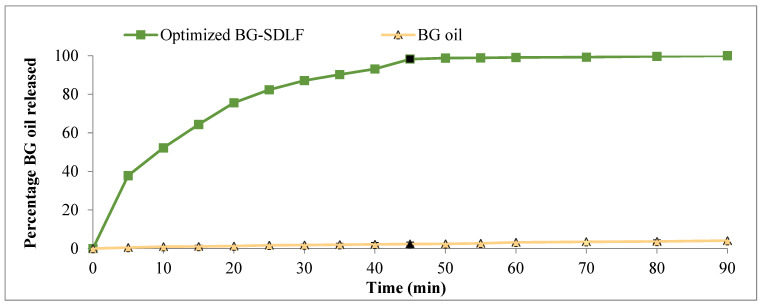
In vitro release profiles of the optimized BG-SDLF against BG oil.

Unlike conventional dosage forms, from which the drug substance simply dissolves in the aqueous dissolution test media, lipid-based formulations release the drug from an oily solution which is often immiscible with water. In vitro dissolution studies are performed to ensure the quick release of the drug into the dissolution medium and give an idea about the self-microemulsification efficiency of the developed system. The release profiles of the optimized BG-SDLF and BG oil were studied as depicted graphically in Figure 4. All determinations were carried out in triplicate, and data are presented as percentage of drug dissolved (mean ± SD) at different time intervals. BG oil (2.31 ± 0.99%) showed a significantly lower percentage of drug dissolved after Q_45min_ while the optimized BG-SDLF showed a percentage of 98.23 ± 0.115%; these outcomes could be attributed to the presence of the BG in the form of a solubilized nanosized droplets leading to enhanced dissolution and possessing a lower emulsification time in addition to an increased surface area [24].

#### 2.3.4. Determination of Surface Morphology by TEM

**Figure 5:**
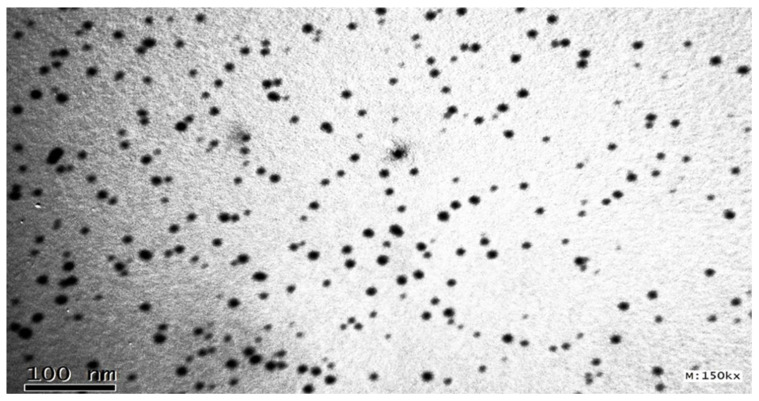
Transmission electron microscopy of the optimized BG-SDLF.

The topography and the globule size of the optimized SDLF-BG were found to be less than 25 nm with discrete and nonaggregated, uniformly shaped spheres after dilution, as depicted in Figure 5.

### 2.4. Biochemical Investigation of Cardioprotective Effect of BG Oil, the Optimized BG-SDLF, and Omega 3 in ISO-Treated Rats

#### 2.4.1. Effect of BG Oil, the Optimized BG-SDLF, and Omega 3 on Plasma BNP and NT-pro-BNP

BNP and NT-proBNP (brain natriuretic peptide) are commonly employed as major indications for the clinical diagnosis of heart failure (HF) and cardiac dysfunction [25,26]. Many studies have shown that BNP and NT-proBNP can be used in extensive animal experiments and postmortem specimens to reflect cardiac function, and that they may additionally serve as postmortem markers in forensic medicine to identify cardiac dysfunction [27,28]. Plasma BNP and NT-pro-BNP levels revealed a significant increase in group II’s animals of 207.34% and 372.51%, respectively, as compared with normal group I, at *p* < 0.01 (Table 2). Moreover, the administration of BG oil (100 mg/kg) (group III) resulted in a significant decrease in plasma BNP and NT-pro-BNP levels of 24.74% and 45.83%, respectively, compared with group II, at *p* < 0.01. Additionally, it showed a significant decrease in plasma BNP and NT-pro-BNP levels of 38.5% and 63.45%, respectively, in the optimized BG-SDLF (group IV) compared with group II (*p* < 0.01), which was better than in the BG oil group. Furthermore, BNP and NT-pro-BNP levels decreased significantly by 42.38% and 57.3%, respectively, in group V compared with group II (*p* < 0.01).

In the present study, we evaluated the cardioprotective activity of BG oil and the optimized BG-SDLF in ISO-induced cardiac remodeling. The study confirmed that BG oil and the optimized BG-SDLF mediated cardiac protection by inhibiting BNP and NT-pro-BNP, decreasing apoptosis, and reducing cardiac fibrosis, which are involved in their pharmacologic effects, with superior results for the optimized BG-SDLF. Our results agree with previous findings that have shown that PUFAs could decrease BNP and NT-proBNP levels [29,30].

#### 2.4.2. Effect of BG Oil, the Optimized BG-SDLF, and Omega 3 on Plasma Cystatin C, Galectin-3, Lp-PLA2, and MMP2 Levels

In Table 3, a significant increase in plasma cystatin c, galectin-3, Lp-PLA2, and MMP2 levels of 282.14%, 188.1%, 148.5%, and 419.59%, respectively, was clearly detected in group II animals in contrast to the normal group (I) at *p* < 0.01. The administration of BG oil (100 mg/kg) resulted in a significant decrease in plasma cystatin c, galectin-3, Lp-PLA2, and MMP2 levels of 34.17%, 28.91%, 17.06%, and 34.97%, respectively, compared with group II, at *p* < 0.01. Furthermore, it was shown that there was a significant decrease in plasma cystatin c, galectin-3, Lp-PLA2, and MMP2 levels of 47.67%, 49.18%, 26.77%, and 64.68%, respectively, in the optimized BG-SDLF group compared with group II (*p* < 0.01). This revealed that the optimized BG-SDLF was more effective at reducing these biomarkers of cardiac damage than BG oil. Moreover, cystatin c, galectin-3, Lp-PLA2, and MMP2 levels decreased significantly by 37.31%, 39.77%, 24.20% and 46.01%, respectively, in group V compared with group II (*p* < 0.01).

Results in this study showed increased levels of cystatin c, galectin-3, Lp-PLA2, and MMP2 in heart disease. Galectin-3, which is mostly expressed in activated macrophages and pathologically injured cardiomyocytes, is thought to have a role in cardiac remodeling, particularly myocardial fibrogenesis, and the development of HF. Galectin 3 causes fibroblast proliferation and heterogeneous collagen deposition, eventually resulting in heart function loss [31,32,33]. Goncalves et al. [34] found that the degradation and disordered synthesis of the extracellular matrix due to increased MMP2 and a decreased activity of endogenous tissue inhibitors led to collagen deposition and oxidative stress [34]. Lp-PLA2 is a potential inflammatory marker of CHF, and its inhibition ameliorates vascular inflammation and slows the progression of atherosclerosis. Ge et al. [35] found that increased plasma level of Lp-PLA2 was associated with an increased risk of CHD. Cystatin has been implicated as a prognostic maker in CVS and is a promising risk marker in patients hospitalized for acute heart failure [36]. The present study suggested that the increased levels of cystatin c, galectin-3, Lp-PLA2, and MMP2 in rats treated with BG oil could be attributed to its high content of saponins, alkaloids, polyphenols, and terpenoids and to its antioxidant effects.

#### 2.4.3. Effect of BG Oil, the Optimized BG-SDLF, and Omega 3 on Plasma cTnI and cTnT Levels

The present data showed a significant increase in plasma cTnI and cTnT levels to 211.76% and 196.42%, respectively, in group II animals as compared with normal group I, (*p* < 0.01). The administration of BG oil (100 mg/kg) resulted in a significant decrease in plasma cTnI and cTnT levels of 27.77% and 28.18%, respectively, compared with group II (*p* < 0.01). Additionally, there was a significant decrease in plasma cTnI and cTn T levels of 50.0% and 43.63%, respectively, in the optimized BG-SDLF compared with group II (*p* < 0.01) with a larger improvement than in the BG oil group. Furthermore, cTnI and cTnT levels decreased significantly by 47.22% and 37.30%, respectively, in group V compared with group II (*p* < 0.01) (Table 4). Our recent data highlighted the elevation of plasma cTnI and cTnT levels was associated with CVD in ISO-treated rats. The present results agreed with other studies that proved the relation between the levels of cTnI and cTnT and CVD [37,38].

#### 2.4.4. Effect of BG Oil, the Optimized BG-SDLF and Omega 3 on Plasma miRNA-21 and miRNA-23a Genes

**Figure 6:**
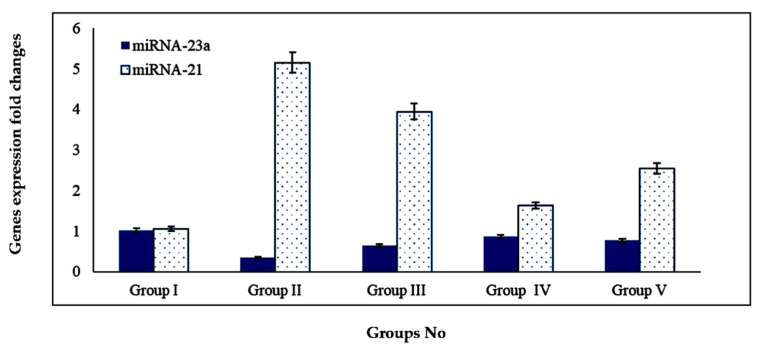
Impact of BG oil and the optimized BG-SDLF on plasma miRNA-23a and miRNA-21 genes’ expression in isoproterenol-treated rats. All groups within each gene expression are obviously different from each other at *p* < 0.05.

In Figure 6, a significant increase in plasma miRNA-21 gene expression level of 490.47% and a significant decrease in plasma miRNA-23a gene expression level of 66.70% were detected obviously in group II animals compared with the normal group I, (*p* < 0.01). The administration of BG oil (100 mg/kg) resulted in a significant decrease in plasma miRNA-21 gene expression level of 23.49%, and a significant elevation in plasma miRNA-23a gene expression level of 191.20% compared with group II, (*p* < 0.01).

Furthermore, it was showed that there was a significant decrease in plasma miRNA-21 gene expression levels of 68.34%, as well as a significant increase in plasma miRNA-23a gene expression level of 252.94% in the optimized BG-SDLF compared with group II (*p* < 0.01), reflecting better results than in the BG oil group. Group V showed a significantly decreased miRNA-21 gene expression level of 50.67%, as well as a significantly increased miRNA-23a gene expression level of 229.41% compared with group II (*p* < 0.01). The results demonstrated the biological roles of miRNA-21 in group II as it was found to be overexpressed in group II as compared with normal rats. However, BG oil and the optimized BG-SDLF administration ameliorated the level of miRNA-21 gene expression due to the presence of high contents of n-3 PUFA [4]. The upregulation of the miRNA-21 gene in CVD cases has been proved in other studies [39].

All major types of cardiovascular cells, including vascular smooth muscle cells (VSMCs), endothelial cells, cardiomyocytes, and cardiac fibroblasts, have been reported to have significant levels of miRNA-21 expression. miRNA-21 is unusually overexpressed in numerous cardiovascular disorders. Furthermore, both loss-of-function and gain-of-function techniques have identified miRNA-21 as playing essential roles in various cardiovascular illnesses in recent research, and the identification of possible target genes for miR-21-mediated cardiovascular effects has begun [40,41,42].

Mandal and colleagues [43] previously demonstrated that a relatively low dose of n-3 PUFA alone could suppress miRNA-21 expression after 24 h via a nuclear factor kappa-B (NF-B)-dependent mechanism. miRNA-21 possesses an NF-B-binding site in its promoter, and earlier research has demonstrated that NF-B regulates miRNA-21, using transfection experiments to show that NF-B is at least partially involved in miRNA-21 transcription. miRNAs other than miRNA-21 may have either hindered or increased the PUFA action on miRNA-21, resulting in varied expression of miRNA-21 across time periods and treatments [44].

The modulation of miRNA-21 had a considerable effect on matrix metalloprotease-2 (MMP-2) expression via its target chromosome 10 (PTEN) [45]. It is worth noting that MMP-2’s biological functions are not confined to fibrosis. MMP-2, in fact, has been identified as a crucial protease in a variety of oxidative-stress-related diseases, including heart failure, atherosclerosis, aneurysm pathogenesis, and myocardial ischemia–reperfusion [46,47].

Moreover, we observed that the level of miRNA-23a expression in group II was downregulated compared with the normal group. miRNA-23a has been implicated in tumor-necrosis-factor-induced protection of bone-marrow-derived mesenchymal stem cell death via regulating caspase-7 and myocardial infarction [48].

Our findings showed that ISO-induced oxidative stress is a frequent mechanism of cardiovascular damage caused by a variety of causes. During the pathological phase of several cardiovascular diseases, such as atherosclerosis, hypertension, ischemic heart disease, and hyperlipidemia, an excessive oxygen free radical formation and reduction of the antioxidant defense mechanism for free radical management have been observed. Our study suggested that BG oil and the optimized BG-SDLF containing n-3 PUFA and polyphenolics caused an upregulation of miRNA-23a in ISO-treated rats. Our results are in line with Li et al. [49], who reported a downregulation of miRNA-23a gene expression in acute myocardial infarction patients. The present study hypothesizes that the cardioprotective effect of BG oil and the optimized BG-SDLF against ISO-induced cardiac toxicity is due to the presence of natural anti-inflammatory and antioxidant ingredients that inhibit the production of BNP, NT-pro-BNP, cystatin c, galectin-3, Lp-PLA2, MMP2, cTnI, and cTnT and cause a downregulation of miRNA-21 as well as an upregulation of miRNA-23a gene expression.

## 3. Materials and Methods

### 3.1. Materials

Bottle gourd (*Lageneria siceraria*) oil (BG oil) was obtained from a cold press, purchased from a local herbalist, (Ministry of Agriculture and Land Reclamation, Agriculture Research Center, Cairo, Egypt); isopropyl myristate (IPM) (Sigma-Aldrich Co., Schnelldorf, Germany); Tween 80 (Merck-Schuchardt, Darmstadt, Germany); Cremophor^®^ RH40 (BASF, Ludwigshafen, Germany). Albino rats, weighing 160 ± 10 g, were obtained from the National cancer institute, Cairo University, and were housed individually in an air-conditioned room at 22 ± 1 °C, with a relative humidity of 60%, and an 8:00-to-20:00 light cycle.

### 3.2. Methods

#### 3.2.1. Preparation of BG Oil Self-Dispersing Lipid Formulations (BG-SDLFs)

Preliminary screening showed a good solubility of BG oil in IPM, Tween 80, and Cremophor^®^ RH40. These components were utilized successfully in the formulation of microemulsions and self-emulsification systems in previous studies [50,51]. BG-SDLFs were formulated using 10% of BG in all formulations. A full 3^2^ factorial design was constructed via Design-Expert software (Version 11.1.2.0, USA) to study the impacts of formulation variables on the characteristics of the prepared BG-SDLFs. Oil concentration (20, 30, and 40% *w*/*w*) and the surfactant mixture ratio of Tween 80/Cremophor^®^ RH40 (T 80: Cr RH40) (2:1, 1:1, and 1:2 *w*/*w*) were set as the independent variables (X_1_ and X_2_, respectively), while DS (Y_1_), PDI (Y_2_), ET (Y_3_), and Tr% (Y_4_) were set as dependent variables as recorded in Table 5.

The significance of the effect of each formulation factor was checked using an analysis of variance (ANOVA), where *p* < 0.05 indicated significance. The best-fitting mathematical model was determined based on the comparison of the multiple correlation coefficient (R^2^) and the predicted and adjusted R^2^ values. Nine formulations were prepared at different formulation variables and the impacts of these variables on the responses were recorded in Table 1. The preparation of BG-SDLFs was carried out by blending the specified weights of each component in a glass vial, followed by a gentle vortex mixing for about 5 min till a homogeneous mixture was obtained. The resultant BG-SDLFs were kept for 3 days at ambient temperature to attain equilibrium before evaluation.

#### 3.2.2. Characterization of the Prepared BG-SDLFs

##### Droplet Size Analysis (DS) and Poly Dispersity Index (PDI) Determination

A diluted dispersion of each formulation was formulated by blending 0.1 mL of each formulation with 10 mL of double-distilled water and stirring well before analysis. DS and PDI were measured via Zetasizer (ZS, Malvern, UK) [52].

##### Emulsification Efficiency Determination

The performance of the self-emulsification of BG-SDLFs was visually evaluated by measuring the time mandatory for the complete dispersion [13,17,53] of 0.5 g of each formulation in 500 mL of 0.1 N HCl at 37 ± 0.5 °C using a dissolution apparatus (USP II, Distek, 2500, North Brunswick, NJ, USA) rotated at 100 rpm. The transmittance percent for each formed formulation dispersion was measured at 638 nm via a spectrophotometer (UV-1800, Shimadzu, Kyoto, Japan) against distilled water as blank [53].

#### 3.2.3. Optimization of BG-SDLFs

The selection of the optimized formula was obtained by applying the desirability function via Design-Expert software (Version 11.1.2.0, USA) to obtain the desired responses criteria listed in Table 5 (minimum DS, PDI, ET, and maximum Tr%). The optimized formulation, with the highest desirability figure, was selected for further evaluation.

#### 3.2.4. Characterization of the Optimum BG-SDLF

##### Visual Inspection, Drug Content Measurement, and Thermodynamic Stability Assessment

The optimized BG-SDLF was visually evaluated for clarity, consistency, and phase separation against strong light. For measurements of drug content, a certain weight of the formulation (0.1 g) was dissolved in 100 mL of methylene chloride for 15 min under continuous stirring. BG oil concentration was measured by a UV spectrophotometer (UV-1800, Shimadzu, Kyoto, Japan) at the predetermined λ_max_ = 276.80 nm. For the assessment of thermodynamic stability, two stress tests were performed: a centrifugation test (3000 rpm for 30 min) and a freezing/thawing cycle test (3 complete cycles, each of freezing for 24 h at −5 °C, followed by thawing for 24 h at 25 °C). Then, each test was visually examined for any turbidity or phase separation.

##### Robustness to Dilution

The optimized BG-SDLF was diluted 50, 100, and 1000 times with various vehicles including distilled water, 0.1 N HCl (pH 1.2), and pH 7.4 phosphate buffer to mimic a physiological dilution after oral administration. The formed dispersions were kept for 2 h, then visually evaluated for transparency and homogeneity [53].

##### Surface Morphology via Transmission Electron Microscopy [TEM]

The diluted BG-SDLF optimum formula was stained with a 2% phosphotungstic acid solution, then placed on a 200-mesh copper grid followed by drying, and photographing via TEM (JEOL, JEM-1230, Tokyo, Japan) [54].

##### In Vitro Dissolution of BG-SDLF Formula

Dissolution studies of the optimized BG-SDLF and BG oil were conducted via a rotating-paddle USP dissolution tester at 50 rpm and 37 ± 0.5 °C in 500 mL of 0.1 N HCl [55]. A sample of 3 mL BG-SDLF (containing 10% BG oil) and an equivalent amount of BG oil (0.3 mL) were placed separately in the dialysis membrane (12,000–14,000 MW cutoff, Sigma-Aldrich, St. Louis, MO, USA) previously soaked for 24 h in 0.1 N HCl, then sealed from both sides by the aid of sutures. This assembly was then placed in a dissolution vessel and the study was performed for 90 min [14]. At appropriate time intervals (5, 10, 15, 20, 25, 30, …, 90 min), a sample of 2 mL was taken, filtered by membrane filter, and analyzed for its BG oil concentration at the predetermined λ_max_ = 277 nm [56] against 0.1 N HCl. All determinations were carried out in triplicates and comparisons were done at 45 min (Q_45min_).

#### 3.2.5. In Vivo Assessment

The in vivo assessment of BG oil and the optimized BG-SDLF to mitigate isoproterenol-evoked cardiac toxicity in rats was carried out using the following tests. The in vivo assessment was performed in accordance with the international standards and the ethical guidelines on animal welfare and was approved by the institutional ethics committee of the faculty of pharmacy, O6U University. Protocol code: 20220802; date of approval: 2 August 2022.

##### Experimental Design

Fifty adult albino rats were housed and acclimated to standard humidity and a natural light–dark cycle. The rats had unlimited access to standard pellets and water. The rats were divided randomly into five groups, each of ten rats. Group I received a regular diet for 30 days, P.O. Group II was fed a regular diet with ISO (85 mg/kg) SC on day 29 and day 30 [57]. Group III was fed a regular diet with BG oil (100 mg/kg) P.O. for 30 days + ISO (85 mg/kg) SC on day 29 and day 30. Group IV was fed a regular diet with the optimized BG-SDLF (100 mg/kg) P.O. for 30 days + ISO (85 mg/kg) SC on day 29 and day 30. Group V was fed a regular diet with omega-3 fatty acids (100 mg/kg) P.O. for 30 days + ISO (85 mg/kg) SC on day 29 and day 30.

##### Determination of BNP, NT-proBNP, cTnI, and cTnT Levels

The estimation of the BNP, NT-pro-BNP, cTnI, and cTnT levels was performed in fresh plasma using the kit manufacturer’s instructions (Abcam, Cambridge, UK).

##### Determination of Cystatin C, Galectin-3, Lp-PLA2, and MMP2 Levels

Cystatin C and galectin-3 levels were determined using rat ELISA kits based on the manufacturer’s instructions (Jiangsu Microplate Biotechnology Company (Changzhou, China) and BG Medicine (Waltham, MA, USA), respectively). Additionally, plasma levels of Lp-PLA2 and MMP2 were measured using commercial ELISA kits: the Lp-PLA2 assay kit (Kangerke Biotech Co., Ltd., Tianjin, China) and an immunoassay ELISA kit (Atlanta, GA, USA), respectively, at a λ_max_ of 450 nm via a spectrophotometer.

##### Quantitative Real-Time PCR for the Determination of miRNA-21 and miRNA-23a

The total RNA of the heart tissues was extracted using a Sepasol-RNA1Super as directed by the manufacturer (Nakarai Tesque, Kyoto, Japan), and portions of the isolated RNA (10–15 g) were subjected to a real-time PCR quantitative analysis. A two-step RT-PCR was used to measure gene expression. MiRNA-21 and miRNA-23a levels were determined using quantitative real-time PCR. The PCR mixture of the reaction included a PCR buffer, 1.5 mM of MgCl_2_, 0.2 mM of each deoxyribonucleotide triphosphate (dNTP), and 0.4 mM of specific primers:

- miRNA-21 primer sequence: F: 5′-ACGTTGTGTAGCTTATCAGACTG-3′

             R: 5′-AATGGTTGTTCTCCACACTCTC-3′

- miRNA-23a primer sequence: F: 5′-GGGGGGGGATCACATTGCCA-3′

             R: 5′-CAGTGCAGGGTCCGAGGT-3′

- U6 (qRT-PCR internal control): F: 5′-GCTTCGGCAGCACATATACTAAAAT-3′

              R: 5′-CGCTTCACGAATTTGCGTGTCAT-3′.

Assays were carried out in a reaction mixture with a volume of 50 mL. Reaction conditions consisted of a preincubation at 50 °C for 2 min and 95 °C for 10 min, followed by 40 cycles of 95 °C for 15 s and 60 °C for 1 min. The values were automatically documented. Quantitative data of the RT-PCR are presented as a percentage of the control. The internal control used was U6 mRNA (one of the most widely used internal reference genes for miRNA).

The obtained outcomes are presented as average ± SD for ten separate spectrophotometric determinations and ELISA measurements as well as for the PCR analysis of genes’ expression. All the data were analyzed by SPSS software (version 20) via an ANOVA followed by Bonferroni’s multiple comparison test at *p* < 0.01.

## 4. Conclusions

BG-SDLFs were successfully prepared using IPM and Tween 80/Cremophor^®^ RH40 in different ratios according to a 3^2^ full factorial design. Formulation factors showed a significant impact on different formulation parameters, and the optimized formula was selected using the desirability function. BG oil and the optimized BG-SDLF provided cardioprotection against ISO-induced cardiac remodeling by inhibiting oxidative-stress-associated inflammation through regulating miRNA-23a and miRNA-21 genes’ expression, BMP, and MMP2 with boosted results for the optimized BG-SDLF. The findings of this study suggest that miRNA-21/miRNA-23a could be used as therapeutic targets in the treatment of cardiotoxicity in clinical studies. Future in vivo and clinical investigations are required for assessing the therapeutic efficiency of BG oil and its formulations.

## Figures and Tables

**Table 1:** Compositions of BG-SDLFs of the 3^2^ factorial design and the measured responses; DS, PDI, ET, and Tr (%).

Formula Code	BG-SDLFs Compositions *			Responses *			
	IPM (% *w*/*w*)	S_mix_ (% *w*/*w*)	T 80/Cr ratio	DS (nm)	PDI	ET (min)	Tr (%)
F1	10	80	2:1	22.6 ± 0.3	0.254 ± 0.009	8.8 ± 0.8	99.5 ± 0.1
F2	10	80	1:1	31.2 ± 0.4	0.192 ± 0.010	7.2 ± 0.5	99.5 ± 0.1
F3	10	80	1:2	29.9 ± 0.1	0.138 ± 0.006	12.0 ± 0.2	99.1 ± 0.3
F4	20	70	2:1	27.1 ± 0.6	0.177 ± 0.044	6.9 ± 0.2	99.6 ± 0.3
F5	20	70	1:1	29.4 ± 0.1	0.136 ± 0.010	6.2 ± 0.4	99.8 ± 0.1
F6	20	70	1:2	33.5 ± 0.3	0.129 ± 0.018	7.4 ± 0.2	99.3 ± 0.1
F7	30	60	2:1	84.3 ± 0.8	0.435 ± 0.025	5.2 ± 0.3	94.8 ± 0.1
F8	30	60	1:1	75.4 ± 1.5	0.335 ± 0.037	6.3 ± 0.8	96.2 ± 0.1
F9	30	60	1:2	54.4 ± 0.1	0.201 ± 0.009	7.2 ± 0.2	98.3 ± 0.1

* All formulations contain 10% bottle gourd oil. Data are expressed as mean ± SD (*n* = 3).

**Table 2:** Impact of BG oil and the optimized BG-SDLF on plasma BNP and NT-pro-BNP in isoproterenol-treated rats.

Group No.	Group Description	BNP (pg/mL)	NT-pro-BNP (pg/mL)
I	Normal control	85.77 ± 7.30 ^a^	21.43 ± 2.19 ^a^
II	Isoproterenol (85 mg/kg)	177.84 ± 9.92 ^b^	79.83 ± 5.7 ^b^
III	Isoproterenol + BG oil (100 mg/kg)	128.50 ± 6.86 ^c^	43.24 ± 5.46 ^c^
IV	Isoproterenol + the optimized BG-SDLF (100 mg/kg)	109.36 ± 16.61 ^d^	29.17 ± 4.80 ^d^
V	Isoproterenol + omega 3 (100 mg/kg)	102.47 ± 10.54 ^d^	34.08 ± 3.53 ^e^

Data are shown as mean ± SD of the numbers of outcomes within each treatment. Data followed by the same letter are not significantly different at *p* ≤ 0.05.

**Table 3:** Impact of BG oil and the optimized BG-SDLF on plasma cystatin c, galectin-3, Lp-PLA2, and MMP2 in isoproterenol-treated rats.

Group No.	Group Description	Cystatin C (mg/L)	Galectin-3 (ng/mL)	Lp-PLA2 (ng/mL)	MMP2 (mg/mL)
I	Normal control	0.84 ± 0.02 ^a^	42.71 ± 3.53 ^a^	327.20 ± 22.75 ^a^	4.90 ± 0.30 ^a^
II	Isoproterenol (85 mg/kg)	2.37 ± 0.21 ^b^	80.34 ± 5.35 ^b^	485.90 ± 30.63 ^b^	20.56 ± 1.86 ^b^
III	Isoproterenol + BG oil (100 mg/kg)	1.56 ± 0.08 ^c^	62.80 ± 6.13 ^c^	402.97 ± 20.75 ^c^	13.37 ± 0.57 ^c^
IV	Isoproterenol + optimized BG-SDLF (100 mg/kg)	1.24 ± 0.19 ^c^	44.89 ± 2.41 ^a^	355.82 ± 27.48 ^d^	7.26 ± 0.37 ^d^
V	Isoproterenol + omega 3 (100 mg/kg)	1.49 ± 0.06 ^c^	53.21 ± 3.64 ^d^	368.31 ± 30.21 ^e^	11.10 ± 1.46 ^c^

Data are shown as mean ± SD of the numbers of outcomes within each treatment. Data followed by the same letter are not significantly different at *p* ≤ 0.05.

**Table 4:** Impact of BG oil and the optimized BG-SDLF on cTnI and cTnT in isoproterenol-treated rats.

Group No.	Group Description	cTnI (ng/mL)	cTnT (ng/mL)
I	Normal control	0.34 ± 0.05 ^a^	0.56 ± 0.06 ^a^
II	Isoproterenol (85 mg/kg)	0.72 ± 0.06 ^b^	1.10 ± 0.09 ^b^
III	Isoproterenol + BG oil (100 mg/kg)	0.52 ± 0.04 ^c^	0.79 ± 0.07 ^c^
IV	Isoproterenol + optimized BG-SDLF (100 mg/kg)	0.36 ± 0.02 ^d^	0.62 ± 0.03 ^d^
V	Isoproterenol + omega 3 (100 mg/kg)	0.38 ± 0.03 ^d^	0.69 ± 0.04 ^e^

Data are shown as mean ± SD of the numbers of outcomes within each treatment. Data followed by the same letter are not significantly different at *p* ≤ 0.05.

**Table 5:** Summary of levels of full factorial design (3^2^) for independent variables, dependent variables, constraints for the optimization of BG-SDLFs, the factors levels for the optimized BG-SDLF, and its predicted responses.

Factors (Independent Variables)	Levels			Optimized Level *
X_1_: Oil concentration (%)	20	30	40	30
X_2_: T 80: Cr RH40 ratio	2:1	1:1	1:2	1:1
Responses (Dependent variables)	Formulation optimization Desirability constraints			Predicted values
Y_1_: DS (nm)	Minimize			29.5
Y_2_: PDI	Minimize			0.141
Y_3_: ET (min)	Minimize			5.9
Y_4_: Tr (%)	Maximize			99.8

* At a desirability of 0.916.

## Data Availability

Data are available upon request from authors.

## References

[1] World Health Organization (2021). Cardiovascular Diseases [CVDs]. https://www.who.int/news-room/fact-sheets/detail/cardiovascular-diseases-[cvds].

[2] Ravassa S., González A., Bayés-Genís A., Lupón J., Díez J. (2020). Myocardial Interstitial Fibrosis in the Era of Precision Medicine. Biomarker-Based Phenotyping for a Personalized Treatment. Rev. Esp. Cardiol..

[3] Hu H., Jiang M., Cao Y., Zhang Z., Jiang B., Tian F., Feng J., Dou Y., Gorospe M., Zheng M. (2020). HuR Regulates Phospholamban Expression in Isoproterenol-Induced Cardiac Remodelling. Cardiovasc. Res..

[4] Abd El-Rahman A.A., Mahmoud A.Z., Sayed A.A., Abd El Latif M.A. (2022). Physiochemical Properties and Phytochemical Characteristics of Bottle Gourd (*Lagenaria siceraria*) Seed Oil. Egypt J. Chem..

[5] Saeed M., Khan M.S., Amir K., Bi J.B., Asif M., Madni A., Kamboh A.A., Manzoor Z., Younas U., Chao S. (2022). Lagenaria Siceraria Fruit: A Review of Its Phytochemistry, Pharmacology, and Promising Traditional Uses. Front. Nutr..

[6] Dhakad G., Tambe K., Shirsat S., Jaiswal N. (2022). Review on Study of Bottle Gourd on Human Health. Res. J. Pharmacol. Pharmacodyn..

[7] Deshpande J.R., Choudhari A.A., Mishra M.R., Meghre V.S., Wadodkar S.G., Dorle A.K. (2008). Beneficial Effects of Lagenaria Siceraria [Mol.] Standley Fruit Epicarp in Animal Models. Indian J. Exp. Biol..

[8] Panchal C.V., Sawale J.A., Poul B.N., Khandelwal K.R. (2013). Hepatoprotective Activity of Lagenaria Siceraria [Molina] Standley Fruits Against Paracetamol Induced Hapatotoxicity in Mice. Int. J. Pharm. Sci. Res..

[9] Saboo S.S., Thorat P.K., Tapadiya G.G., Khadabadi S.S. (2013). Ancient and Recent Medicinal Uses of Cucurbitaceae Family. Int. J. Ther. Appl..

[10] Gershanik T., Benita S. (2000). Self-Dispersing Lipid Formulations for Improving Oral Absorption of Lipophilic Drugs. Eur. J. Pharm. Biopharm..

[11] Kumar S., Kumar Gupta S., Kumar Sharma P. (2012). Self-Emulsifying Drug Delivery Systems [SEDDS] for Oral Delivery of Lipid Based Formulations. African J. Basic Appl. Sci..

[12] Ameta R.K., Soni K., Bhattarai A. (2023). Recent Advances in Improving the Bioavailability of Hydrophobic/Lipophilic Drugs and Their Delivery via Self-Emulsifying Formulations. Colloids Interfaces.

[13] Ujilestari T., Martien R., Ariyadi B., Danar Dono N., Zuprizal (2018). Self-nanoemulsifying Drug Delivery System [SNEDDS] of Amomum Compactum Essential Oil: Design, Formulation, and Characterization. J. Appl. Pharm. Sci..

[14] El-Haddad A.E., Sheta N.M., Boshra S.A. (2018). Isolation, Formulation, and Efficacy Enhancement of Morin Emulsified Carriers Against Lung Toxicity in Rats. AAPS PharmSciTech.

[15] El-Mancy S.S., El-Haddad A.E., Alshareef W.A., Saadeldeen A.M., El-Emam S.Z., Elnahas O.S. (2021). Enhancement of Antimicrobial and Antiproliferative Activities of Standardized Frankincense Extract Using Optimized Self-Nanoemulsifying Delivery System. Sci. Pharm..

[16] Abdelbari M.A., El-Mancy S.S., Elshafeey A.H., Abdelbary A.A. (2021). Implementing Spanlastics for Improving the Ocular Delivery of Clotrimazole: In Vitro Characterization, Ex vivo Permeability, Microbiological Assessment and In vivo Safety Study. Int. J. Nanomed..

[17] Cuiné J.F., Charman W.N., Pouton C.W., Edwards G.A., Porter C.J.H. (2007). Increasing the Proportional Content of Surfactant [Cremophor EL] Relative to Lipid in Self-emulsifying Lipid-based Formulations of Danazol Reduces Oral Bioavailability in Beagle Dogs. Pharm. Res..

[18] Date A.A., Nagarsenker M.S. (2007). Design and evaluation of self-nanoemulsifying drug delivery systems [SNEDDS] for cefpodoxime proxetil. Int. J. Pharm..

[19] Elsayed I., El-Dahmy R.M., Elshafeey A.H., El Gawad N.A.A., El Gazayerly O.N. (2019). Tripling the Bioavailability of Rosuvastatin Calcium Through Development and Optimization of An In-Situ Forming Nanovesicular System. Pharmaceutics.

[20] Taha E., Samy A., Kassem A., Khan M. (2005). Response Surface Methodology for the Development of Self-Nanoemulsified Drug Delivery System [SNEDDS] of All-Trans-Retinol Acetate. Pharm. Dev. Technol..

[21] Wang L., Dong J., Chen J., Eastoe J., Li X. (2009). Design and optimization of a new self-nanoemulsifying drug delivery system. J. Colloid. Interface Sci..

[22] Fayez S.M., Elnahas O.S., Fayez A.M., El-Mancy S.S. (2023). Coconut Oil Based Self-Nano Emulsifying Delivery Systems Mitigate Ulcerogenic Nsaids Side Effect and Enhance Drug Dissolution: Formula Optimization, In-Vitro, and In-Vivo Assessments. Int. J. Pharm..

[23] Sanka K., Suda D., Bakshi V. (2016). Optimization of Solid-Self Nanoemulsifying Drug Delivery System for Solubility and Release Profile of Clonazepam Using Simplex Lattice Design. J. Drug Deliv. Sci. Technol..

[24] Balata G.F., Essa E.A., Shamardl H.A., Zaidan S.H., Abourehab M.A.S. (2016). Self-Emulsifying Drug Delivery Systems as a Tool to Improve Solubility and Bioavailability of Resveratrol. Drug Des. Devel. Ther..

[25] Maalouf R., Bailey S. (2016). A Review on B-type Natriuretic Peptide Monitoring: Assays and Biosensors. Hear. Fail. Rev..

[26] Cocco G., Jerie P. (2015). Assessing the Benefits of Natriuretic Peptides-Guided Therapy in Chronic Heart Failure. Cardiol. J..

[27] Palmiere C., Tettamanti C., Bonsignore A., De Stefano F., Vanhaebost J., Rousseau G., Scarpelli M.P., Bardy D. (2018). Cardiac Troponins and NT-Probnp in The Forensic Setting: Overview of Sampling Site, Postmortem Interval, Cardiopulmonary Resuscitation, and Review of The Literature. Forensic. Sci. Int..

[28] Michaud K., Augsburger M., Donzé N., Sabatasso S., Faouzi M., Bollmann M., Mangin P. (2008). Evaluation of Postmortem Measurement of NT-Probnp as a Marker for Cardiac Function. Int. J. Legal. Med..

[29] Berger R., Huelsman M., Strecker K., Bojic A., Moser P., Stanek B., Pacher R. (2002). B-Type Natriuretic Peptide Predicts Sudden Death in Patients with Chronic Heart Failure. Circulation.

[30] De Groote P., Dagorn J., Soudan B., Lamblin N., McFadden E., Bauters C. (2004). B-type Natriuretic Peptide and Peak Exercise Oxygen Consumption Provide Independent Information for Risk Stratification in Patients with Stable Congestive Heart Failure. J. Am. Coll. Cardiol..

[31] Ibarrola J., Arrieta V., Sádaba R., Martinez-Martinez E., Garcia-Peña A., Alvarez V., Fernández-Celis A., Gainza A., Santamaría E., Fernández-Irigoyen J. (2018). Galectin-3 Down-Regulates Antioxidant Peroxiredoxin-4 in Human Cardiac Fibroblasts: A New Pathway to Induce Cardiac Damage. Clin. Sci..

[32] Ibarrola J., Sádaba R., Garcia-Peña A., Arrieta V. (2018). A Role for Fumarate Hydratase in Mediating Oxidative Effects of Galectin-3 in Human Cardiac Fibroblasts. Int. J. Cardiol..

[33] MacKinnon A.C., Farnworth S.L., Hodkinson P.S., Henderson N.C., Atkinson K.M., Leffler H., Nilsson U.J., Haslett C., Forbes S.J., Sethi T. (2008). Regulation of Alternative Macrophage Activation by Galectin-3. J. Immunol..

[34] Gonçalves P.R., Nascimento L.D., Gerlach R.F., Rodrigues K.E., Prado A.F. (2022). Matrix Metalloproteinase 2 as a Pharmacological Target in Heart Failure. Pharmaceuticals.

[35] Ge P.C., Chen Z.H., Pan R.Y., Ding X.Q., Liu J.Y., Jia Q.W., Liu Z., He S.Z., An F.H., Li L.H. (2016). Synergistic Effect of Lipoprotein-Associated Phospholipase A2 with Classical Risk Factors on Coronary Heart Disease: A Multi-Ethnic Study in China. Cell. Physiol. Biochem..

[36] Lassus J., Harjola V., Siirilawaris K., Sund R., Melin J., Pulkki K., Peuhkurinen K., Nieminen M.S. (2006). 643 Prognostic Value of Cystatin C in Acute Heart Failure in Relation to Other Markers of Renal Function and NT-proBNP. Eur. J. Hear. Fail. Suppl..

[37] Welsh P., Preiss D., Shah A.S.V., Mcallister D., Briggs A., Boachie C., McConnachie A., Hayward C., Padmanabhan S., Welsh C. (2018). Comparison between High-Sensitivity Cardiac Troponin T and Cardiac Troponin I in a Large General Population Cohort. Clin. Chem..

[38] Neumann J.T., Havulinna A.S., Zeller T., Appelbaum S., Kunnas T., Nikkari S., Jousilahti P., Blankenberg S., Sydow K., Salomaa V. (2014). Comparison of Three Troponins as Predictors of Future Cardiovascular Events—Prospective Results from the FINRISK and BiomaCaRE Studies. PLoS ONE.

[39] Sekuklu S.D., Donoghue M.T.A., Spillane C. (2009). miR-21 As A Key Regulator of Oncogenic Processes. Biochem. Soc. Trans..

[40] Cheng Y., Ji R., Yue J., Yang J., Liu X., Chen H., Dean D.B., Zhang C. (2007). MicroRNAs are Aberrantly Expressed in Hypertrophic Heart: Do They Play a Pole in Cardiac Hypertrophy?. Am. J. Pathol..

[41] Suárez Y., Fernández-Hernando C., Pober J.S., Sessa W.C. (2007). Dicer Dependent MicroRNAs Regulate Gene Expression and Functions in Human Endothelial Cells. Circ. Res..

[42] Ji R., Cheng Y., Yue J., Yang J., Liu X., Chen H., Dean D.B., Zhang C. (2007). MicroRNA Expression Signature and Antisense-Mediated Depletion Reveal an Essential Role of Microrna in Vascular Neointimal Lesion Formation. Circ. Res..

[43] Mandal C.C., Ghosh-Choudhury T., Dey N., Choudhury G.G., Ghosh-Choudhury N. (2012). miR-21 is Targeted by Omega-3 Polyunsaturated Fatty Acid to Regulate Breast Tumor CSF-1 Expression. Carcinogenesis.

[44] Xiao Y., Xu C., Guan J., Ping Y., Fan H., Li Y., Zhao H., Li X. (2012). Discovering Dysfunction of Multiple MicroRNAs Cooperation in Disease by a Conserved MicroRNA Co-Expression Network. PLoS ONE.

[45] Roy S., Khanna S., Hussain S.-R.A., Biswas S., Azad A., Rink C., Gnyawali S., Shilo S., Nuovo G.J., Sen C.K. (2009). MicroRNA Expression in Response to Murine Myocardial Infarction: Mir-21 Regulates Fibroblast Metalloprotease-2 via Phosphatase and Tensin Homologue. Cardiovasc. Res..

[46] Schulz R. (2007). Intracellular Targets of Matrix Metalloproteinase-2 in Cardiac Disease: Rationale and Therapeutic Approaches. Annu. Rev. Pharmacol. Toxicol..

[47] Viappiani S., Nicolescu A., Holt A., Sawicki G. (2009). Activation and modulation of 72 kDa matrix metalloproteinase-2 by Peroxynitrite and Glutathione. Biochem. Pharmacol..

[48] Mao J., Lv Z., Zhuang Y. (2014). MicroRNA-23a is involved in tumor necrosis factor-α induced apoptosis in Mesenchymal Stem Cells and Myocardial Infarction. Exp. Mol. Pathol..

[49] Li S., Ren J., Sun Q. (2018). The Expression of MicroRNA-23a Regulates Acute Myocardial Infarction in Patients and In Vitro Through Targeting PTEN. Mol. Med. Rep..

[50] Nazeam J.A., Ragab G.M., El-Gazar A.A., El-Mancy S.S., Jamil L., Fayez S.M. (2021). Topical Nano Clove/Thyme Gel against Genetically Identified Clinical Skin Isolates: In Vivo Targeting Behavioral Alteration and IGF-1/pFOXO-1/PPAR γ Cues. Molecules.

[51] Badawi A.A., ABD EL-Aziz N., Amin M.M., Sheta N.M. (2014). Topical Benzophenone-3 Microemulsion-Based Gels: Preparation, Evaluation and Determination of Microbiological UV Blocking Activity. Int. J. Pharm. Pharma. Sci..

[52] Batool A., Arshad R., Razzaq S., Nousheen K., Kiani M.H., Shahnaz G. (2020). Formulation and Evaluation of Hyaluronic Acid-Based Mucoadhesive Self Nanoemulsifying Drug Delivery System [SNEDDS] of Tamoxifen for Targeting Breast Cancer. Int. J. Biol. Macromol..

[53] Gupta S., Chavhan S., Sawant K.K. (2011). Self-Nanoemulsifying Drug Delivery System for Adefovir Dipivoxil: Design, Characterization, In Vitro and Ex Vivo Evaluation. Colloids Surfaces a Physicochem. Eng. Asp..

[54] Soliman S.M., Sheta N.M., Ibrahim B.M.M., El-Shawwa M.M., Abd El-Halim S.M. (2020). Novel Intranasal Drug Delivery: Geraniol Charged Polymeric Mixed Micelles for Targeting Cerebral Insult as a Result of Ischaemia/Reperfusion. Pharmaceutics.

[55] Ibrahim T., El-Megraba N., Abdallaha M. (2018). Self-Emulsifying Drug Delivery Formulations. Zagazig J. Pharm. Sci..

[56] Atta N., Ismaiel G.H.Z. (2020). Usage of Oil and Powder of Bottle Gourd and Pumpkin Seeds in Production of High Nutritive Value Biscuit. Egypt J. Nutr. Health.

[57] Boshra S.A. (2020). Resveratrol Modulates miR-34a in Cardiotoxicity Induced by Isoproterenol. J. Med. Food..

